# A proprietary blend of standardized *Punica granatum* fruit rind and *Theobroma cocoa* seed extracts mitigates aging males' symptoms: A randomized, double-blind, placebo-controlled study

**DOI:** 10.7150/ijms.73645

**Published:** 2022-07-11

**Authors:** Sucharitha L. Pandit, Dayanand Yaligar, Manjunath Halemane, Annapoorna Bhat

**Affiliations:** 1 Shetty's Hospital, Kaveri Nagar, Bengaluru-560068, Karnataka, India.; 2 Narayana Hrudayalaya, Bommasandra Industrial Area, Bengaluru-560068, Karnataka, India.; 3 D2L Clinical Solutions, Sadaramangala Industrial Area, Bengaluru-560048, Karnataka, India.; 4 Sri Venkateshwara Hospital, Madiwala, Bengaluru-560068, Karnataka, India.

**Keywords:** Aging males' symptoms, LN18178, Muscular strength, Tesnor^®^, Testosterone, Stress alleviation

## Abstract

**Objective**: We evaluated the safety and efficacy of a novel combination of *Punica granatum* fruit rind and *Theobroma cocoa* seed extracts (LN18178 or Tesnor^®^) in enhancing serum testosterone level and reducing aging males' symptoms (AMS) in a randomized, double-blind, placebo-controlled investigation (CTRI Reg. No. CTRI/2019/02/017506).

**Methods:** One hundred twenty healthy male participants (age 36-55 years) were randomized (n=40) to placebo, 200 or 400 mg of LN18178 for a period of fifty-six consecutive days of supplementation. The primary efficacy parameter was the AMS score. The secondary parameters were serum testosterone (free and total) levels, hand-grip strength, and perceived stress scale (PSS-10) score. Measurement of other hormones included in the study was serum dihydrotestosterone (DHT), cortisol, and 17β-estradiol (E2). Total blood chemistry parameters, vital signs, and urinalysis were parts of the safety assessment.

**Results:** Both doses of LN18178 significantly reduced the mean AMS scores after 56 days of supplementation. Furthermore, it significantly improved general, psychological, and sexual well-being. Serum levels of free testosterone and total testosterone levels were significantly increased in LN18178 supplemented (200 and 400 mg) participants compared to the baseline and placebo. Significant improvements in hand-grip strength and reduced PSS-10 scores were also observed.

**Conclusion:** LN18178 supplementation reduced AMS scores and improved sexual performance. Also, LN18178 groups exhibited superior muscular strength and reduction in perceived stress. Total blood chemistry and urine analysis demonstrated the broad-spectrum safety.

## Introduction

Advancing age is a physiological progression associated with structural and functional decline in many human body organs and complemented by adaptations in behavior, physical activity, body composition, and reductions in sexual energy and endocrine functions 1. Aging males' symptoms (AMS) include declining physical (somatic), psychological and sexual functions with advancing age in men. During aging, men suffer from a gradual decline in androgen level, which is known as androgen deficiency in the aging male (ADAM) or late-onset hypogonadism (LOH) 2. Testosterone, the principal androgen of the male reproductive system, is essential for the regulation of several physiological functions, including sexual arousal, development of secondary sexual characteristics, bone and muscle mass, muscular strength, production of red blood cells (RBC), metabolic homeostasis, healthy sperm profile, psychological wellness 3, 4. In men, testosterone levels decline with age 5. In the '30s, men start declining serum testosterone levels of around 1 - 1.4% per year as testosterone synthesis and bioavailability of free testosterone (FT) decline with advancing age. Total testosterone (TT) level drops at an average of 1.6% per year, while FT level declines by 2-3% in men after 50 years of age 6. A decline in circulating testosterone level also reduces lean body mass (LBM), muscular strength, and physical performance 4. Low testosterone level decreases aging adults' physical performance, mood status, energy level, and quality of life (QOL). A low level of FT causes erectile dysfunction, loss of muscle mass, reduced sexual desire, abnormal abdominal fat gain, low bone mineral density, joint pain, sleep disturbances, fatigue, depression, and a decline in cognitive functions 7-9. Since ancient times, selected phytonutrients have demonstrated enhanced muscle mass, muscle strength, and sexual function 10. Indian folklore medicine comprehends many plants, including *Tribulus terrestris, Mucuna prurines, Withania somnifera,* and their extracts for general well-being and to increase male hormone level, improve sexual function, penile erection, and spermatogenesis 10, 11.

LN18178 or Tesnor^®^ is a standardized, proprietary blend of *Punica granatum* (PG) fruit rind and *Theobroma cocoa* seed (TC) extracts. Recently, in a fifty-six-day human trial, LN18178 significantly increased serum TT and FT in concurrence with increased muscle strength and size in healthy young males 12. This herbal blend increases steroidogenesis via activating CYP17A1 in mouse Leydig cells *in vitro* (unpublished observation). *Punica granatum* L., commonly known as the pomegranate, is a long-living deciduous shrub native of Iran, widely cultivated in the Mediterranean region, Southeast Asia, and tropical Africa 13. The fruit rind has various pharmacological activities such as antioxidant, immune-modulatory, anti-diabetic, anti-plasmodial, anti-microbial, wound healing, anti-hyperglycemic, hepatoprotective, and anti-diarrheal 14, 15. Peels of pomegranate fruit were also used to treat infection of male and female sexual organs, acne, mastitis, piles, allergic dermatitis, and timpanists 15. *Theobroma cocoa*, also known as Cocoa beans, is a widely used raw material in chocolate confectioneries. Cocoa is a tropical tree that originated from the headwaters of the Amazon River, a tropical American region 16. Polyphenolic antioxidants in Cocoa provide several health benefits, including protection against hypertension, endothelial dysfunction, inflammation, platelet activation 17, 18.

The objective of the present fifty-six-day trial was to assess the efficacy of LN18178 (Tesnor^®^) supplementation on AMS, muscular strength, serum testosterone levels in aging males. Furthermore, we have evaluated the changes in other hormones in the participants. This study also demonstrates that this botanical blend is tolerable to the study participants.

## Materials and methods

### Study material

LN18178 (Tesnor^®^) is a standardized herbal blend combining aqueous ethanol extracts of *Punica granatum* fruit rind (PG) and *Theobroma cocoa* seed (CT) at a 4:1 ratio. The mixture was formulated into a free-flowing powder with 25% excipients. The final product was standardized to contain at least 3.5% punicalagins and 0.5% theobromine. LN18178 is a patent-pending proprietary composition developed at Laila Nutraceuticals R&D Centre, Vijayawada. Detailed descriptions of the plant raw materials, extraction procedures, and phytochemical analysis using High Performance Liquid Chromatography (HPLC) are mentioned earlier 12.

### Study Design

The present randomized, double-blind, placebo-controlled clinical trial was conducted to assess the influence of LN18178 on aging males' symptoms and circulating male hormones, including testosterone in healthy aging males (Age: 36 to 55 yrs). Three independent sites in Bengaluru, Karnataka, India (Shetty's Hospital, Sri Venkateswara Hospital, and Narayana Hrudayalaya Hospital) conducted this study following the ICH-GCP guidelines. Individual ethics committees of these three study centers reviewed and approved the study protocol. The trial was registered (CTRI/2019/02/017506) in the Clinical Trial Registry of India (CTRI).

### Study Participants

One hundred and twenty men [age: 36-55 years; body mass index (BMI): 20-29 kg/m^2^] participated in this study. The participants had aging males' symptoms (AMS) scores between 27 and 43. They expressed their willingness to walk for 30 minutes per day (five days a week) and not to start any therapies for sexual health, consumption of energy supplements, protein supplements, or health drinks during the study. The participants were recruited through a preliminary screening via telephone. The inclusion and exclusion criteria for recruiting the study participants are summarized in **Table 1**. All subjects were aware of this study's potential risks/benefits. The participants read and signed the informed consent form (ICF).

### Randomization, blinding, and supplementation

**Figure 1:**
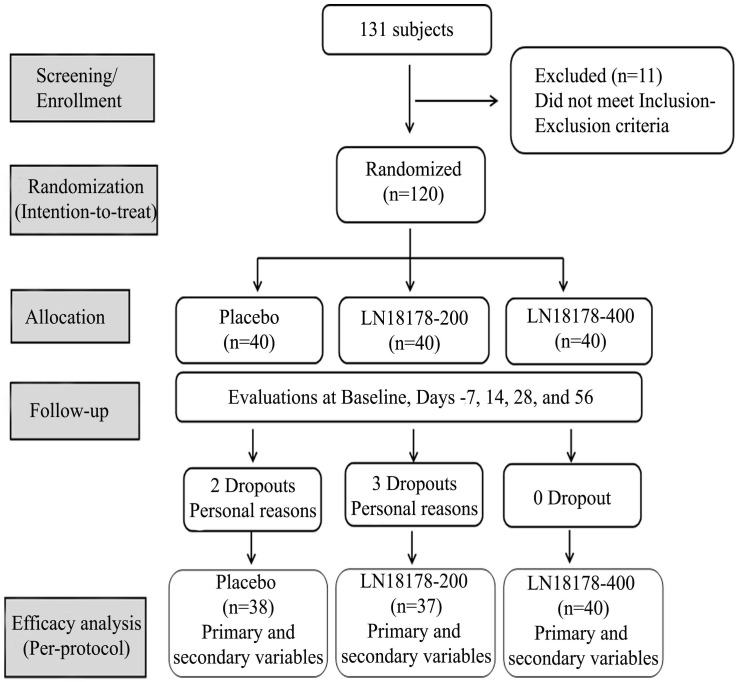
A CONSORT diagram summarizes the flow of the study, participants' enrollment, follow-up, and analysis.

One hundred and twenty eligible subjects were recruited and randomly allocated into three groups (n=40) viz. LN18178-200 mg, LN18178-400 mg or placebo through computer-generated block randomization using PROC-PLAN procedure in SAS (SAS Institute Inc.). Investigational products (IP) were packed in high-density polyethylene (HDPE) bottles identical in size and appearance. IP bottles were marked in a sequence. Once enrolled in the trial, participants were supplied with the following available number in a randomly allocated IP bottle. Investigators and the study team were blinded to the randomization. As advised, each participant consumed one hard gelatin capsule of study product (placebo or LN18178-200 mg or LN18178-400 mg) after breakfast over a period of fifty-six consecutive days. Placebo capsules were identical to LN18178 capsules in color, size, weight, and appearance. The study consisted of screening, randomization, baseline visits, and four follow-up visits on days 7, 14, 28, and 56 (**Figure 1**). Study site coordinators contacted the participants over the telephone about their adherence to study protocols throughout the study duration. Adverse event monitoring was strictly enforced.

### Primary efficacy measure

#### Aging males' symptoms score

Change of aging males' symptoms (AMS) score from baseline was the primary efficacy measure of the present study. AMS is a simple and effective measure to characterize a decline in general well-being with low testosterone levels in men. AMS consists of a 17-item self-reported questionnaire distributed into three sub-scales, psychological (7 questions), somatic (5 questions), and sexual (5 questions), with a maximum total score of 85 and a minimum of 17 19. Each item is scored from 1 to 5 depending on the response, with 5 being the most severe. Participants completed the AMS questionnaire at baseline, on days 7, 14, 28, and 56 of supplementation. Twenty milliliters of venous blood samples were collected from the overnight fasted participants between 6.30 and 7.30 AM at each visit. The clotted blood samples were centrifuged (350 × *g* for 15 min) at 4 °C. The serum samples were stored in aliquots at -20 °C until analyses.

### Secondary efficacy measure

#### Serum hormone analysis

We determined the serum hormones viz. total testosterone (TT), free testosterone (FT), 17β-estradiol (E2), dihydrotestosterone (DHT), and cortisol to evaluate the efficacy of LN18178 at baseline, day 7, 14, 25, and 56 of supplementation. The serum levels of creatinine (Cr), blood urea nitrogen (BUN), aspartate transaminase (AST), alanine transaminase (ALT), and alkaline phosphatase (ALP) were measured at baseline and end of the study. Hormone analysis was performed using commercially available specific and sensitive EIA kits. Ortho Clinical Diagnostics (Raritan, NJ, USA) supplied TT, cortisol, and E2 assay kits. The FT and DHT kits were procured from Diagnostics Biochem Canada Inc. (London, ON, Canada) and BioVendor - Laboratorní medicína a.s. (Karásek, Czechia), respectively. All assays were performed following the manufacturer's protocols. The hormone concentrations were calculated using the standard curves from each assay. The analytical sensitivities of FT, TT, DHT, Cortisol, and E2 assay kits were 0.062 pmol/L, 0.17 nmol/L, 0.080 nmol/L, 2.83 nmol/L, and 23.347 pmol/L, respectively.

#### Hand-grip Strength

Hand-grip strength of the participants was determined using a digital dynamometer (INCO Instruments & Medical Devices Pvt. Limited, Ambala, India) as an indicator of muscle strength at baseline and follow-up visits. Participants were asked to extend their forearm of the dominant hand on a table and allowed to bend at 90° with the elbow. The dynamometer was gently placed on the hand of the participant and asked to squeeze as hard as possible without any jerking motion. Three measurements were taken at 2 min intervals, and the best performance was recorded.

#### Perceived Stress Scale

The perceived stress scale (PSS) is a validated measure of the perception of stress, comprising of a 10-item questionnaire. Each item is scored from 0 to 4, and the total PSS score ranges from 0 to 50 20. The participants completed the questionnaire at baseline and all follow-up visits.

#### Safety parameters

As a part of the safety assessment, total blood chemistry, including a series of hematological, serum biochemical measurements, and urinalysis, were performed at screening and the end of the study. VITROS® 5600 integrated system (Dry Chemistry analyzer, Ortho Clinical Diagnostics, New Jersey, USA) was used for blood chemistry and urine analysis. The clinical chemistry parameters were fasting glucose, serum creatinine, uric acid, blood urea nitrogen (BUN), serum bilirubin and alanine transaminase (ALT), aspartate aminotransferase (AST), alkaline phosphatase (ALP), sodium, potassium, serum albumin. The hematology parameters were hemoglobin, platelet count, total leukocyte count, RBC, ESR, differential count. Urinalysis included color, specific gravity, pH, glucose, protein, and RBC. Microscopic examinations were performed under a clinical light microscope (Olympus Opto Systems India Pvt. Ltd. New Delhi, India). Participants' vital signs, including blood pressure (systolic/diastolic), pulse rate, respiratory rate, and oral temperature, were recorded at all study visits.

#### Sample size calculation and power analysis

Approximately 36 subjects per treatment group were assumed to provide 90% power to detect a treatment effect in the primary efficacy variable at a two-sided significance level of 0.05%. A mean effect size of 1.20 and a deviation of 1.10 were considered in the sample size calculation based on an earlier study on an herbal supplement 21. With an assumption of 10% dropout, 120 subjects (40 subjects per arm) were recruited in this study.

### Statistical analysis

The data are expressed as mean ± SD. The efficacy parameters were tested for significance using Student's t-test for 'within-the-group' and ANCOVA for 'between-the-group' comparisons. A one-way ANOVA was used for evaluating baseline characteristics, vital signs, and laboratory parameters. A p-value of <0.05 was considered statistically significant. Statistical analyses were performed using the SAS 9.4 software (SAS Institute, Inc, Cary, NC).

## Results

The present study enrolled one hundred and twenty healthy men (age: 36-55 y) with aging males' symptoms (AMS) scores between 27 and 43. Participants consumed one capsule of either placebo or LN18178 200 mg or LN18178 400 mg after breakfast for fifty-six consecutive days. The mean age of participants was 45.90 ± 6.283, 43.66 ± 5.809, and 45.20 ± 5.459 years in the placebo, LN18178 200 mg, and LN18178 400 mg groups, respectively. Baseline demographics were comparable among the study groups and are presented in **Table 2**. Five subjects discontinued the study because of their personal reasons. A total of 115 subjects completed the study, and the data was analyzed in the per-protocol (PP) population.

**Table 3** presents the gradual improvements in the total AMS scores and the scores of AMS sub-scales from baseline to follow-up visits. An early and significant reduction in total AMS scores was observed in LN18178 400 mg group starting from day 7 (P < 0.05) from baseline and placebo, through the end of the trial (p<0.05 vs. baseline and placebo). In the LN18178-200 mg group, improvements in total AMS scores were significant from day 14 through the end of the trial. After 56 days of supplementation, the total AMS scores were significantly (P < 0.05 vs. baseline and placebo) decreased by 15.51% and 19.30% in LN18178 200 and 400 mg groups, respectively. In placebo, the total AMS score was reduced by only 4.55% at the end of supplementation (**Table 3**).

The AMS subscale score analyses reveal that 56 days of supplementation of low and high dose LN18178 resulted in significant reductions in psychological behavior (10.95% and 11.51%), somatic behavior (19.54% and 25.14%), and sexual behavior (12.65% and 15.83%) from baseline. In contrast, the changes in these subscale scores in the placebo group were minimal and not significant compared to baseline (**Table 3**).

**Table 4** shows gradual improvements in the hand-grip strength of the LN18178-supplemented participants during the study. The low and high-dose herbal supplemented groups showed 18.95% and 24.64% increases in hand-grip strength from baseline. An early and significant increase (P < 0.05) in the muscle strength in both LN18178 groups started from day 7 of supplementation.

From baseline, the mean PSS scores in LN18178 200- and 400 mg groups started significant reductions from day 14 through the end of the study. Following 56 days of supplementation, the mean decreases (P < 0.05) in PSS scores were 15% and 23% in 200- and 400 mg groups, respectively, compared to placebo (**Table 4**).

### Efficacy of LN18178 on serum hormones

Increases in free testosterone (FT) and total testosterone (TT) during 56 consecutive days of LN18178 supplementation are presented in **table 5**. LN18178 supplementation significantly increased the FT level by 39.16% and 48.28%; the TT level by 21.39% and 24.56% in 200- and 400 mg groups, respectively, from baseline. The LN18178 400 mg group showed early and significant increases in FT and TT levels from baseline following seven days of supplementation. Compared to placebo, increases in FT and TT levels were substantial in 200- and 400 mg LN18178 groups.

LN18178 supplementation did not significantly change the E2 levels in the participants (**Table 5**). However, post-trial, the high-dose LN18178 group showed 23.54% (P < 0.05; vs. baseline) and 31.13% (P < 0.05; vs. placebo) increases of the TT and E2 (TT/E2) ratio (**Table 5**).

At the end of the study, the LN18178 supplemented groups did not significantly alter their serum cortisol and Dihydrotestosterone (DHT) levels compared to baseline and placebo (**Table 5**).

### Safety parameters

The routine hematology, clinical chemistry parameters, and the participants' vital signs were unaltered during the trial and remained within the normal laboratory ranges (data not shown). Also, the metabolic markers, Cr, AST, ALT, ALP, and BUN, were within the normal ranges during the trial (**Table 6**). Overall, the participants reported only five minor adverse events: fever, cough & cold, constipation, and diarrhea during the study (**Table 7**).

## Discussion

The present human study demonstrates the efficacy and safety of LN18178, a proprietary blend of *Punica granatum* (PG) rind and *Theobroma cocoa* (TC) seed extracts in healthy men (age: 36-55 yrs.) with mild to moderate aging 'males' symptoms (AMS) scores. Recent human clinical investigations revealed the potential benefits of botanical and nutraceutical supplementation in reducing the severity of AMS scores and boosting testosterone levels in aging males 21-24. This is the first report demonstrating the benefits of a standardized, herbal blend of PG rind and TC seed extracts (LN18178) in improving AMS, TT, FT, and muscular strength in aging men. PG is traditionally known as a symbol of fertility 25. Also, the juice of the fruit and its pericarp are employed to relieve colic, colitis, menorrhagia, headache, and other conditions in traditional medicine, demonstrating a broad spectrum safety of PG 14, 26. PG rind powder and extracts have extensive application in the dairy industry 27. Cocoa beans are widely used in confectionaries and are rich in potent antioxidants 28. Earlier, a ninety-day repeated dose oral toxicity study in rats and genetic toxicological studies have established a broad-spectrum safety of LN18178. This sub-chronic toxicity study estimated the no-observed-adverse-effect level (NOAEL) of LN18178 in male and female rats was 2500 mg/kg body weight (BW), which is greater than 26 g/day in human of an average BW of sixty-five kg 29. This estimated human equivalent dose is at least 65-fold greater than the higher dose in the present trial. Increases in TT and FT levels and muscle strength in the aging subjects of the present study corroborate with the earlier observation that LN18178 supplementation increased serum testosterone levels associated with increased muscle strength and size in young males 12.

Testosterone replacement therapy is the primary treatment option for correcting the androgen deficiency in males 30, 31. However, because of possible increased risk of heart attacks and strokes, US FDA recommends a restriction of testosterone therapy for certain medical conditions or the subjects with testicular dysfunction or disorder in gonadal-hypophyseal axis, rather than age related androgen deficiency 32. Interestingly, in the recent past, a series of botanical products have been shown to increase testosterone levels in young or aging/older males 33. Rao et al demonstrated that *Trigonella foenum-graecum* seed extract increased testosterone levels and age related symptoms including sexual function in healthy aging males 22. Further, supplementation of different preparations of *Withania somnifera* extracts increased testosterone level and demonstrated beneficial effects in healthy and infertile men in independent clinical trials 33.

In the present study, fifty-six days of supplementation of LN18178 significantly improved the total AMS scores compared to baseline and placebo. AMS scale is a validated tool in assessing health-related quality of life (HRQoL) and consequences of advancing age in men 34. Early and significant improvements in AMS scores were observed in LN18178 (400 mg group) from day seven to the end of the study. Significant improvements in psychological well-being, somatic behavior, and sexual behavior in the LN18178-supplemented participants are important observations of the present study.

Another interesting observation of this trial was significant improvements in serum testosterone levels in both intervention groups following fifty-six consecutive days of supplementation. Both serum TT and FT levels gradually started increasing significantly from day 14 till the end of the study, which highlights the potential benefits of LN18178 in improving somatic and sexual behavior. An increase in serum testosterone level supports the mechanistic basis for increased testicular steroidogenesis 35, 36. Testosterone plays an essential role in regulating metabolic homeostasis, maintaining muscle and bone integrity, and fat metabolism 37, 38. Increased testosterone level prevents age-related metabolic dysregulation, muscle and bone mass loss, and fat accumulation 39. Also, increased testosterone level improves libido in men 38. An interesting observation from the present study is a significantly increased TT/E2 ratio in the LN18178 supplemented subjects. It is worth noting that a positive correlation exists between the TT/E2 ratio and increased sexual desire, semen quality, and libido in men 40. Therefore, the improved score in the sexual behavior domain of the AMS questionnaire in association with increased testosterone level in the herbal supplemented subjects is noteworthy.

Hand-grip strength is a widely recognized tool to assess muscular isometric strength 41. Significant improvement in hand-grip strength of the participants on either LN18178 200 or 400 mg supplementation highlights the positive correlation between muscular strength and serum testosterone levels 42. Moreover, LN18178 supplemented participants showed a significant stress reduction. This study's observations align with the earlier findings that suggest an improvement in testosterone level attenuates stress and enhances sexual desire in men 43, 44.

The botanical formulation LN18178 is well tolerated by the study participants. The complete blood chemistry analysis, including serum creatinine, BUN, AST, ALT, and ALP levels in LN18178 supplemented subjects are within the physiological range, which further substantiates the broad-spectrum safety of LN18178.

## Conclusion

LN18178 (Tesnor®), a novel botanical proprietary blend of *Punica granatum* fruit rind, and *Theobroma cocoa* seed extracts, is well-tolerated and safe for human consumption. This study suggests that LN18178 attenuates the severity of AMS, boosts serum testosterone levels, and improves the psychological, sexual, and general well-being of healthy aging men. LN18178 also increases muscular strength and reduces stress. LN18178 is a well-tolerated, safe, and effective nutraceutical blend that boosts sexual function, testosterone level, and psychological and general well-being in aging males.

## Figures and Tables

**Table 1:** Inclusion and Exclusion Criteria

Inclusion Criteria	Exclusion Criteria
Male subjects (age 36 - 55 yrs, BMI: 22-29 kg/m^2^	History of taking medications for erectile dysfunction, oligospermia urinary and clinical history of endocrine disorders eg: pituitary tumors, hypo- and hyper-thyroidism.
Subjects with total AMS total between 27 and 43.	Subjects diagnosed with sleep apnea and having history of psychiatric disorder.
Willingness to walk for 30 min/day (5 days/week)	Subjects consuming alcohol or smoking and taken any medications that can interfere with muscle mass such as corticosteroids, testosterone replacement.
Healthy subjects with willing partners and good health	Subjects under medications including anti-hypertensives, inhaled beta agonists, anti-hyperlipidemics, psychotropic and consume recreational drugs( cocaine) etc.
Ability to understand the risks and benefits of the protocol	Subjects having history of Benign Prostate Hyperplasia (BPH), hypertension, diabetes, stroke, angina, life-threatening arrhythmia within the past 6 months.
Subjects should written ICF &available thought out the study	Subjects using anorectic agents and endocannabinoid neuromodulators in last 2 weeks are prohibited.
Subjects agree to maintain the activity diary.	Subjects with HIV positive or any other STDs and underwent major surgical procedures in last 6 months.
Subjects who agree not to start any therapies for sexual health or consuming energy boosting supplements or protein supplements or health drinks during the course of the study.	Subject has illness as per the opinion of investigator.
Subjects willing to refrain from drinking coffee or caffeinated drinks or beverages during the study	Subjects participated in any other clinical studies from last 90 days

**Table 2:** Baseline characteristics of the participants of the study

Variables	Placebo (n=40)	LN18178-200 (n=40); P-value	LN18178-400 (n=40); P-value
Age (y)	45.90 ± 6.28	43.66 ± 5.81; 0.2893	45.20 ± 5.46; 0.8615
Body weight (kg)	73.99 ± 8.25	75.26 ± 8.73; 0.7809	73.37 ± 8.00; 0.9412
BMI (kg/m^2^)	26.11 ± 1.98	25.96 ± 1.85; 0.9508	25.56 ± 2.28; 0.4664
AMS score	36.45 ± 4.02	35.58 ± 4.54; 0.6406	35.13 ± 4.24; 0.3501
Grip strength (Kg)	35.85 ± 11.25	35.56 ± 12.95; 0.9942	35.11 ± 13.16; 0.9625
PSS-10 score	19.90 ± 5.15	20.39 ± 5.25; 0.9092	19.60 ± 5.36; 0.9647
Free Testosterone (pmol/L)	42.68 ± 10.62	41.64 ± 13.39; 0.9949	40.95 ± 11.56; 0.9433
Total Testosterone (nmol/L)	12.62 ± 4.84	12.48 ± 5.47; 0.9997	13.69 ± 5.13; 0.4930
Dihydrotestosterone (nmol/L)	2.22 ± 0.77	2.25 ± 0.90; 0.7981	2.20 ± 0.93; 0.9940
Cortisol (nmol/L)	207.55 ± 79.10	198.44 ± 52.69; 0.8082	221.90 ± 98.26; 0.7033
Estradiol (pmol/L)	90.86 ± 35.29	95.05 ± 30.45; 0.8557	91.46 ± 35.90; 0.9966

Data present as mean ± SD; Intent-to-treat (ITT) population. No significant differences between the placebo and the LN18178 groups; Data were analyzed using ANOVA.

**Table 3:** Effect of LN18178 supplementation on AMS scores of the participants through the course of the trial

Variable	Evaluation days	Placebo	LN18178-200	LN18178-400
AMS score	Baseline	36.26 ± 4.038	35.73 ± 4.507	35.13 ± 4.238
	Day 7	36.11 ± 4.279	35.41 ± 4.456 (0.0698*; 0.8835#)	33.75 ± 4.612 (0.0006*; 0.0037#)
	Day 14	35.75 ± 4.666	34.30 ± 4.795 (0.0005*; 0.2427#)	31.85± 4.666 (<0.0001*; <0.0001#)
	Day 28	35.18 ± 5.082	32.38 ± 4.399 (<0.0001*; 0.0041#)	30.33 ± 4.911 (<0.0001*; <0.0001#)
	Day 56	34.61 ± 5.335	30.19 ± 4.162 (<0.0001*; <0.0001#)	28.35 ± 4.828 (<0.0001*; <0.0001#)
Psychological behavior	Baseline	10.45 ± 1.969	10.05 ± 2.134	9.3 ± 1.977
	Day 7	10.45 ± 1.927	10.08 ± 2.152 (0.5708*; 0.9981#)	9.0 ± 2.013 (0.0087*; 0.0468#)
	Day 14	10.53 ± 2.076	9.92 ± 2.126 (0.4636*; 0.515#)	8.7± 1.937 (0.0034*; 0.0025#)
	Day 28	10.29 ± 2.229	9.59 ± 2.088 (0.0419*; 0.4065#)	8.48 ± 1.811 (0.0009*; 0.0085#)
	Day 56	10.26 ± 2.036	8.95 ±1.84 (<0.0001*; 0.0009#)	8.23 ± 1.544 (0.0001*; <0.0001#)
Somatic behavior	Baseline	17.03 ± 3.209	17.3 ± 3.915	17.3 ± 3.466
	Day 7	16.95 ± 3.337	16.97 ± 3.685 (0.0319*; 0.6651#)	16.38 ± 3.372 (0.0003*; 0.0047#)
	Day 14	16.97 ± 3.69	16.11 ± 3.68 (<0.0001*; 0.1106#)	14.95 ± 3.45 (<0.0001*;0.0001#)
	Day 28	16.34 ± 3.707	14.95 ± 3.448 (<0.0001*; 0.0016#)	14.23 ± 3.206 (<0.0001*; <0.0001#)
	Day 56	15.79 ± 3.974	13.92 ±2.947 (<0.0001*; 0.0013#)	12.95 ± 3.17 (<0.0001*; <0.0001#)
Sexual Behavior	Baseline	8.79 ± 2.591	8.38 ± 2.419	8.53 ± 2.407
	Day 7	8.71 ± 2.567	8.35 ± 2.418 (0.324*; 0.6651#)	8.38 ± 2.549 (0.2047*;0.7958#)
	Day 14	8.58 ± 2.489	8.27 ± 2.502 (0.4573*; 0.9517#)	7.9 ± 2.34 (0.0027*; 0.1082#)
	Day 28	8.55 ± 2.501	7.84± 2.18 (0.0033*; 0.3303#)	7.63 ± 2.295 (0.0003*; 0.0190#)
	Day 56	8.55 ± 2.49	7.32 ± 2.015 (0.0001*; 0.0088#)	7.18 ± 2.308 (<0.0001*; 0.0004#)

Data present as mean ± SD. Placebo (n=38), LN18178-200 (n=37), and LN18178-400 (n=40). In parentheses, * and # indicate P-values in intragroup comparison (vs. baseline) using student t-test and in intergroup comparison (vs. placebo) analyzed using ANCOVA, respectively; NS, not significant.

**Table 4:** Effect of LN18178 supplementation on hand-grip strength and PSS-10 score of the participants through the course of the trial

Variable	Evaluation days	Placebo	LN18178-200	LN18178-400
Hand-Grip Strength (Kg)	Baseline	35.31 ± 11.28	35.52 ± 13.13	35.11 ± 13.16
	Day 7	36.59 ± 13.01	37.45 ± 12.24 (0.0349*; 0.8292#)	38.12 ±14.82 (0.0001*; 0.2627#)
	Day 14	37.12 ± 11.73	39.26 ±12.58 (0.0003*; 0.4544#)	40.96 ±16.14 (0.0003*; 0.0341#)
	Day 28	37.00 ± 13.62	39.94 ± 11.43 (0.0001*; 0.2829#)	42.75 ± 16.18 (<0.0001*; 0.0031#)
	Day 56	36.65 ± 11.19	42.25 ± 12.37 (<0.0001*; 0.0056#)	43.76 ± 15.34 (<0.0001*; <0.0001#)
Perceived Stress Scale (PSS-10) score	Baseline	19.63 ± 5.096	20.14 ± 5.067	19.60 ± 5.363
	Day 7	19.45 ± 5.060	19.92 ± 5.325 (0.0882*; 0.9854#)	19.38 ± 5.348 (0.1068*; 0.9752#)
	Day 14	18.97 ± 4.594	18.68± 4.410 (0.0002*; 0.1851#)	17.85 ± 4.742 (0.0001*; 0.0172#)
	Day 28	18.89 ± 4.637	17.38 ± 4.412 (0.0001*; 0.0031#)	16.25 ±4.749 (0.0001*; <0.0001#)
	Day 56	18.92 ± 5.18	16.14 ± 4.263 (<0.0001*; 0.0003#)	14.50 ± 5.149 (<0.0001*; <0.0001#)

Data present as mean ± SD. Placebo (n=38), LN18178-200 (n=37), and LN18178-400 (n=40). In parentheses, * and # indicate P-values in intragroup comparison (vs. baseline) using student t-test and in intergroup comparison (vs. placebo) analyzed using ANCOVA, respectively; NS, not significant.

**Table 5:** Effect of LN18178 supplementation on serum hormones of the participants

Variable	Evaluation days	Placebo	LN18178-200	LN18178-400
Free Testosterone (pmol/L)	Baseline	42.68±10.62	41.64±13.39	40.95±11.56
	Day 7	41.99 ± 9.44	44.76 ± 12.39 (0.121*; 0.2594#)	45.46 ± 13.29 (0.0193*; 0.1151#)
	Day 14	41.64 ±12.21	48.23 ±16.34 (0.0115*; 0.0452#)	51.01 ± 14.75 (0.0001*; 0.0015#)
	Day 28	42.23 ± 9.79	53.09 ± 16.41 (<0.0001*; 0.0006#)	54.48 ± 15.34 (<0.0001*; <0.0001#)
	Day 56	43.03 ± 11.31	57.95 ± 18.84 (<0.0001*; <.0001#)	60.72 ±17.73 (<0.0001*; <.0001#)
Total Testosterone (nmol/L)	Baseline	12.62 ±4.84	12.48 ±5.47	13.69 ±5.13
	Day 7	11.99 ± 4.50	12.86 ±5.28 (0.4464*; 0.2859#)	14.95 ±6.15 (0.0512*; 0.0287#)
	Day 14	11.93 ± 4.91	13.10 ± 5.01 (0.1564*; 0.066#)	14.56 ± 4.91 (0.0071*; 0.0077#)
	Day 28	12.41 ± 5.94	13.97 ± 4.75 (0.0093*; 0.0642#)	15.49 ± 4.84 (0.0002*; 0.0071#)
	Day 56	12.96 ± 5.32	15.15 ± 6.01 (<0.0001*; 0.0164#)	17.05 ± 4.98 (<0.0001*; 0.0003#)
Dihydrotestosterone (nmol/L)	Baseline	2.22 ± 0.77	2.25 ± 0.90	2.20 ± 0.93
	Day 7	2.17 ± 0.83	2.24 ± 0.97 (0.921*; 0.9257#)	2.12 ± 0.85 (0.2537*; 0.9199#)
	Day 14	2.18 ± 0.85	2.27 ± 0.94 (0.8117*; 0.8478#)	2.10 ± 0.85 (0.2487*; 0.819#)
	Day 28	2.23 ± 0.81	2.32 ± 0.94 (0.4704*; 0.8448#)	2.17 ± 0.88 (0.7256*; 0.9219#)
	Day 56	2.22 ± 0.68	2.32 ± 0.73 (0.3151*; 0.6744#)	2.37 ± 0.75 (0.1021*; 0.2113#)
Cortisol (nmol/L)	Baseline	207.55 ± 79.10	198.44 ± 52.69	221.90 ± 98.25
	Day 7	222.73 ± 69.52	200.65± 60.31 (0.8413*; 0.4793#)	222.45 ± 91.19 (0.9763*; 0.9512#)
	Day 14	203.96 ± 66.54	200.10 ± 71.32 (0.893*; 0.9998#)	216.10 ± 99.86 (0.6984*; 0.9443#)
	Day 28	214.45 ± 76.81	212.79 ± 67.73 (0.2384*; 0.9921#)	211.96 ± 93.37 (0.5651*; 0.8728#)
	Day 56	232.94 ± 81.72	228.25 ± 70.13 (0.0359*; 0.9944#)	236.80 ± 95.41 (0.4001*; 0.9976#)
Estradiol (pmol/L)	Baseline	90.86 ± 35.29	95.05 ± 30.45	91.46 ± 35.90
	Day 56	92.30 ± 27.83	96.99 ± 25.35 (0.6541*; 0.8802#)	93.25 ± 34.79 (0.7694*; 0.9923#)
Testosterone/Estradiol	Baseline	154.90±66.70	132.27±55.53	157.65±71.64
	Day 56	148.52±62.66	161.16±78.53 (0.0718*; 0.4442#)	194.76±78.61 (0.0302*; 0.0012#)

Data present as mean ± SD. Placebo (n=38), LN18178-200 (n=37), and LN18178-400 (n=40). In parentheses, * and # indicate P-values in intragroup comparison (vs. baseline) using student t-test and in intergroup comparison (vs. placebo) analyzed using ANCOVA, respectively.

**Table 6:** Effect of LN18178 supplementation on serum metabolic markers of the study participants

Variable	Evaluation days	Placebo	LN18178-200	LN18178-400
Creatinine (μmol/L)	Baseline	77.79 ± 10.17	82.21 ± 12.02	77.79 ± 13.26
	Day 56	76.02 ± 12.55	83.98 ± 14.14 (0.110*; 0.099#)	78.67 ± 14.76 (0.5808*; 0.4686#)
BUN (mg/dL)	Baseline	9.91 ± 2.96	9.69 ± 2.68	9.38 ± 2.44
	Day 56	9.86 ± 2.93	9.09 ± 1.98 (0.181*; 0.3804#)	10.54 ± 2.48 (0.006*; 0.136#)
AST (U/L)	Baseline	24.13 ± 8.30	21.97 ± 5.75	22.48 ± 6.40
	Day 56	25.16 ± 10.26	23.20 ± 5.85 (0.3661*; 0.3156#)	23.64 ± 6.23 (0.4977*; 0.4287#)
ALT (U/L)	Baseline	27.53 ± 13.20	23.38 ± 12.30	23.65± 8.89
	Day 56	29.70 ± 15.77	24.05 ± 12.35 (0.8164*; 0.0888#)	25.54 ± 13.30 (0.4507*; 0.2108#)
ALP (U/L)	Baseline	86.89±17.90	84.70 ± 20.87	86.23 ± 19.88
	Day 56	85.21 ± 20.23	83.84 ± 20.81 (0.8588*; 0.7729#)	84.65 ± 19.79 (0.6508; 0.9019#)

Data present as mean ± SD. Placebo (n=38), LN18178-200 (n=37), and LN18178-400 (n=40). ALP, alkaline phosphatase, ALT, alanine Transaminase; AST, aspartate aminotransferase, BUN, blood urea nitrogen. In parentheses, * and # indicate P-values in intragroup comparison (vs. baseline) using student t-test and in intergroup comparison (vs. placebo) analyzed using ANCOVA, respectively. The changes are not significant.

**Table 7:** Summary of adverse events reported by the participants during the trial

Adverse events	Placebo (n=38)	LN18178-200 (n=37)	LN18178-400 (n=40)
Fever	1	1	0
Common cold and cough	0	1	0
Constipation	0	0	1
Diarrhoea	1	0	0
Total number of adverse events	2	2	1

## Abbreviations

ALP: Alkaline phosphatase
ALT: Alanine aminotransferase/Glutamic
AMS: Aging males' symptoms
AST: Aspartate aminotransferase
BMI: Body mass index
BUN: Blood urea nitrogen
Cr: Creatinine
DHT: Dihydrotestosterone
E2: 17β- Estradiol
FBS: Fasting blood sugar
HRQoL: Health-related quality of life
ICF: informed consent form
IP: investigational product
MUAC: Mid-upper arm circumferences
PG: *Punica granatum*
TT: total testosterone
FT: Free testosterone
TC: *Theobroma cocoa*
